# Continual Versus Occasional Blood Pressure (COOL-BP) in Remote Hypertension Management

**DOI:** 10.1093/ajh/hpaf003

**Published:** 2025-01-10

**Authors:** Ozan Unlu, Christopher P Cannon, Simin Lee, Daniel Gabovitch, David Zelle, Nicholas Chin, Christian Figueroa, Emma Collins, Ryan Ruggiero, Tiago P Almeida, David Perruchoud, Josep Sola, Benjamin M Scirica, Naomi D L Fisher

**Affiliations:** Accelerator for Clinical Transformation, Mass General Brigham, Boston, MA, USA; Cardiovascular Division, Brigham and Women’s Hospital, Boston, MAUSA; Department of Biomedical Informatics, Harvard Medical School, Boston, MA, USA; Harvard Medical School, Boston, MA, USA; Accelerator for Clinical Transformation, Mass General Brigham, Boston, MA, USA; Cardiovascular Division, Brigham and Women’s Hospital, Boston, MAUSA; Harvard Medical School, Boston, MA, USA; Cardiovascular Division, Brigham and Women’s Hospital, Boston, MAUSA; Harvard Medical School, Boston, MA, USA; Accelerator for Clinical Transformation, Mass General Brigham, Boston, MA, USA; Accelerator for Clinical Transformation, Mass General Brigham, Boston, MA, USA; Accelerator for Clinical Transformation, Mass General Brigham, Boston, MA, USA; Accelerator for Clinical Transformation, Mass General Brigham, Boston, MA, USA; Accelerator for Clinical Transformation, Mass General Brigham, Boston, MA, USA; Accelerator for Clinical Transformation, Mass General Brigham, Boston, MA, USA; Aktiia SA, Neuchâtel, Switzerland; Aktiia SA, Neuchâtel, Switzerland; Aktiia SA, Neuchâtel, Switzerland; Accelerator for Clinical Transformation, Mass General Brigham, Boston, MA, USA; Cardiovascular Division, Brigham and Women’s Hospital, Boston, MAUSA; Harvard Medical School, Boston, MA, USA; Accelerator for Clinical Transformation, Mass General Brigham, Boston, MA, USA; Harvard Medical School, Boston, MA, USA; Division of Endocrinology, Diabetes and Hypertension, Brigham and Women’s Hospital, Boston, MA, USA

**Keywords:** blood pressure, continual blood pressure monitoring, cuffless blood pressure monitor, home blood pressure monitoring, hypertension, remote health

## Abstract

**BACKGROUND:**

Remote hypertension management programs have emerged as potential solutions to improve poor rates of blood pressure (BP) control. The Continual Versus Occasional Blood Pressure (COOL-BP) Study investigated the feasibility and efficacy of using a cuffless wrist BP monitor in a remote hypertension (HTN) program.

**METHODS:**

COOL-BP was a prospective single-arm study within a larger HTN management program at Mass General Brigham (MGB). Participants had uncontrolled HTN, were already engaged in the MGB Remote Hypertension Program, and used a smartphone. The study involved patients wearing the Aktiia cuffless wrist BP monitor and performing traditional home BP monitoring (HBPM). The primary endpoint was the correlation of BP measurements between devices. Secondary endpoints included concordance between HBPM and cuffless pressures following a medication titration, and patient satisfaction with the cuffless device.

**RESULTS:**

We enrolled 38 patients, of whom 25 provided BP data on overlapping dates with both devices. There was moderate correlation between average nonsimultaneous daytime BPs within the same time periods (*r* = 0.57, 95% CI: 0.39–0.71 for systolic BP [*P* < 0.001]; *r* = 0.64, 95% CI: 0.48–0.76, for diastolic BP [*P* < 0.001]). The concordance of systolic BP changes detected by the two devices post medication titration was 87.5%. Most patients (91%) preferred the cuffless device, citing ease of use and convenience.

**CONCLUSIONS:**

Cuffless BP devices demonstrate promise in enhancing patient compliance and effectiveness in HTN management. Their integration into clinical practice could offer a more patient-friendly and reliable approach to BP monitoring, though more research is needed to establish their utility in large populations.

Undertreatment of hypertension (HTN) remains a persistent clinical challenge. Nearly three-quarters of patients with HTN do not have controlled blood pressure (BP), even though many established treatments are generic, widely available, guideline-directed, and cost-effective.^1^ As a potential scalable solution, remote chronic disease management programs have been established to monitor and treat patients with uncontrolled BP.^2^ We have designed and successfully implemented an algorithmically driven Remote HTN Program that uses navigators and pharmacists, supported by specialists, to initiate and titrate medications within the Mass General Brigham health system.^3^

Accurate and consistent measurement of BP continues to be suboptimal, owing to rare use of ambulatory BP monitoring (ABPM) and low adherence to home BP monitoring (HBPM). One survey study showed that less than half of US adults aged 50–80 with HTN routinely measure their BP at home.^4^ With the advent of new technologies and wearables being integrated into medical use, optical BP monitoring technologies have emerged as a potential tool to monitor and manage HTN.^5^ However, the use of these technologies in the remote setting has not yet been studied. We designed the Continual Versus Occasional Blood Pressure (COOL-BP) Study to investigate the feasibility of using a cuffless wrist BP monitor (Aktiia bracelet, Aktiia SA, Neuchâtel, Switzerland) as part of a remote HTN program. Particularly, we investigated its correlation with HBPM, patient preferences for the BP monitoring modality, and its ability to detect BP changes after a medication titration.

## METHODS

### Participants

This study was conducted within the larger Mass General Brigham Remote HTN Program, a remote, algorithmically driven, disease management program that uses navigators and pharmacists, supported by specialists, to initiate and titrate medications within the Mass General Brigham health system. As part of the program, patients with uncontrolled BP were identified through medical record screening or direct referrals. Enrolled patients measured their BP with traditional, digitally connected oscillometric home BP devices. All patients were asked to measure their BP daily for at least 6 days, at baseline and after each medication titration. The guideline-directed measurement schedule requested duplicate readings each morning and evening before medications.^6^ All medication titrations in the management program were based on average weekly home BPs from these standard cuffs.

Participants engaged in the Remote HTN program were identified and invited to join the COOL-BP study. An engaged patient was defined by timely measurement and transmission of BP readings for at least one week following enrollment. Patients had to own a smartphone; full inclusion and exclusion criteria are listed in **Table 1**.

**Table 1.** Inclusion/exclusion criteria

**Inclusion criteria**
Age 26 to 80
Fluent in written and spoken English
Already enrolled in the Remote Hypertension Program
Average of last 3 office blood pressures > 140/90 mm Hg in last 18 months OR Last office blood pressure > 140/90 mm Hg in the last 6 months OR Referred by MD and last blood pressure > 130/80 mm Hg
Owns a smartphone
**Exclusion criteria**
Conditions that limit the use of wrist monitor including tachycardia (heart rate at rest > 120 bpm), persistent atrial fibrillation, Raynaud’s disease, trembling and shivering, arteriovenous fistula, arm amputation, exfoliative skin disease, lymphoedema, known allergy to silicone
Medical comorbidities, including severe heart failure (LVEF < 35%), pheochromocytoma, CKD 4–5 (eGFR ≤ 30 ml/min/1.73m^2^), and any terminal medical condition
Known pregnancy or breastfeeding
Not Massachusetts resident
Last MGB office visit > 3 years or no MGB provider

### Trial design, procedures, and endpoints

COOL-BP was a prospective single-arm study (NCT05211648). Participants who consented to participate in COOL-BP were contacted about the study by a patient navigator who reviewed the protocol and the initialization procedure. They each received a validated, CE-marked, commercially available cuffless wrist BP monitor that measures BP optically (Aktiia SA, Neuchâtel, Switzerland).^7–11^ The Aktiia device is packaged together with its own upper arm oscillometric cuff for once-a-month calibration; clinical trials performed with this monitor have shown that calibrations are reliable for one month.^10^ The device was shipped with written material to instruct the user on how to download the phone app, pair their phone and the bracelet, and initialize the bracelet. Initialization consisted of a series of simultaneous measurements performed by the Aktiia bracelet and the Aktiia upper arm cuff. The procedure was fully automatized and controlled by the Aktiia smartphone application; participants were simply required to sit still while measurements were performed. To supplement the written instructions for patients who required further assistance, a patient navigator called each participant upon arrival of their device shipment and offered to guide them through each step of the set-up.

Once initialization was complete, participants were instructed to wear the bracelet throughout the day and night for the duration of the study. Participants had to recharge the bracelet and synchronize the bracelet data with their smartphone once per week, and reinitialize the device once per month. For up to six months, patients continued to follow procedures for the Remote HTN Program in parallel to wearing the Aktiia Bracelet. This included measuring home BP with a cuff twice in the morning and twice at night for 6 days per week following each medication titration. We defined morning hours as 6 am to 12 pm and evening hours as 6 pm to 12 am. Patients were also asked to fill out an online survey at 30, 60, and 180 days after enrollment.

The primary endpoint of the study was correlation of weekly BP averages provided by bracelet and HBPM. The secondary endpoint was patients’ perception (satisfaction and comfort) of an optical BP monitoring modality compared to an HBPM modality, assessed by surveys. Another secondary endpoint was change in BP tracked by the bracelet compared to HBPM after a medication titration. No significant adverse events were expected from using the Aktiia investigational device.

### Statistical methods and data preparation

A qualifying period of HBPM measurements was defined by at least 12 measurements over at least three days. Since HBPM measurements were obtained only during awake hours, cuffless bracelet measurements only between 6 am and 10 pm were analyzed. The last hour of the day was defined as 10 pm for cuffless measurements, to ensure omission of any sleep BPs. Similar to HBPM, a qualifying period of cuffless bracelet measurements was defined by at least 12 valid bracelet measurements between 6 am and 10 pm over at least three days. Intervals that contained measurements from both qualifying HBPM and the bracelet were identified and tested for correlation between the two modalities. If there were more than two HBPM measurements transmitted in a given morning or evening, only the first pair was included. Importantly, cuffless BPs were not simultaneous with HBPM. They also included measurements in different body positions and were more numerous. The correlation between cuffless BP and HBPM was analyzed using Pearson’s correlation coefficient, with significance set at *P* < 0.05.

For the BP change analysis, a minimum 7-day drug stabilization period was required following each drug titration, during which no recalibration of the cuffless device occurred. This was to ensure observed BP changes were attributed solely to physiological responses and not to any recalibration process. Valid pre-and post-titration data periods were defined as those with at least 12 readings over 3–7 days for HBPM, and at least twelve 24-h readings over the same timeframe for Aktiia. For each BP modality, BP change was calculated as the difference between average BP values within each post-/pretitration data event. By calculating the average BP values before and after titration without the influence of recalibration, we aimed to measure the impact of medication adjustments directly (recalibration-independent analysis of BP changes).

A concordance plot was created to compare systolic BP (SBP) changes measured by both HBPM and the cuffless wrist monitor. For this analysis, a predetermined 4 mmHg range was established as a “no-change zone,” which is less than half of the tolerable error margin for the cuff.^12^ Concordance between the devices was defined when both showed either a rise or a drop, or when both fell within the “no-change zone” for BP changes. The concordance rate was calculated as the ratio of matching readings over the total measurements taken.

## RESULTS

**Figure 1.**
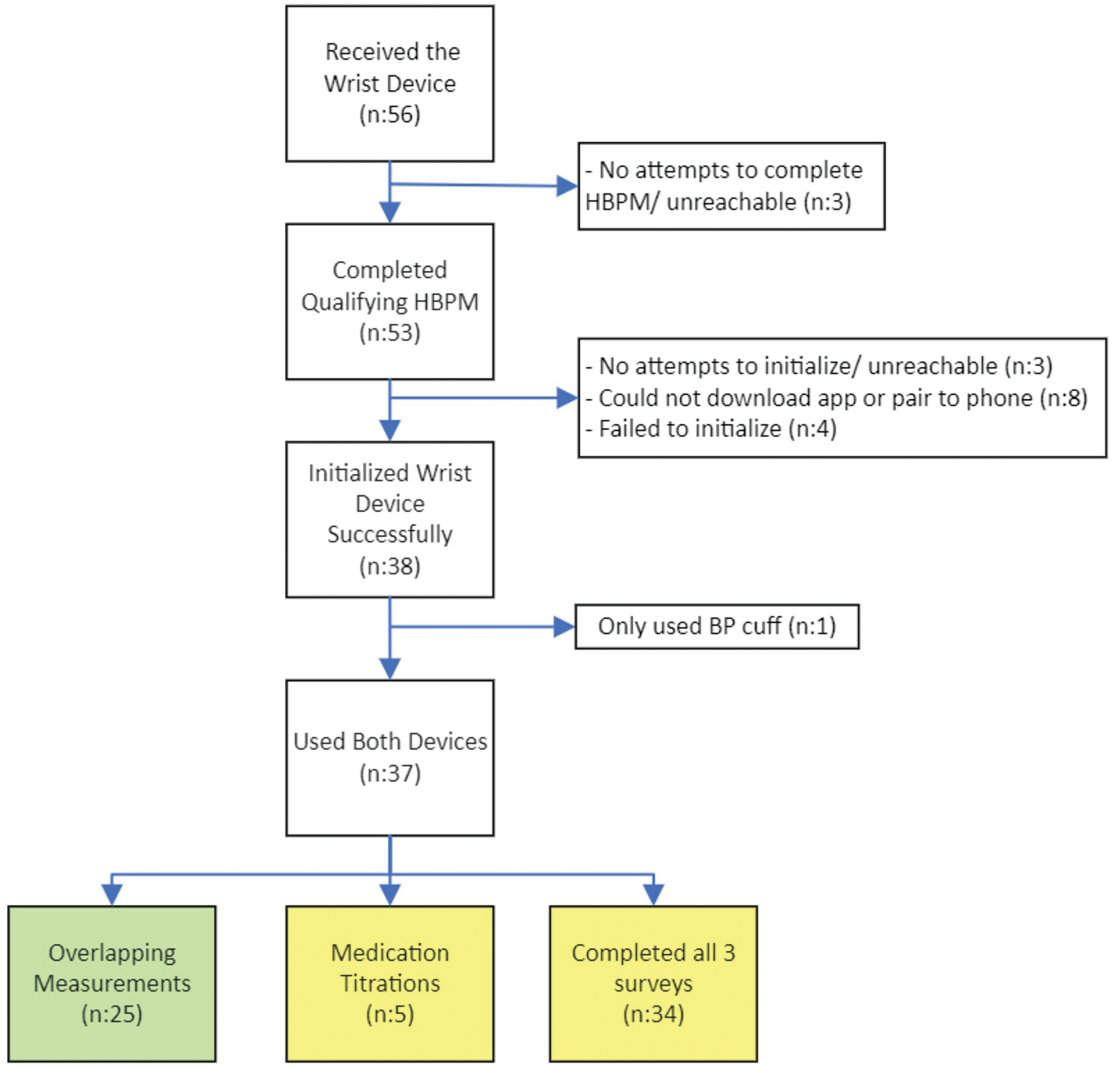
Consort diagram. Consort diagram of cohorts for primary and secondary endpoints. At the bottom of the figure, leftmost box indicates the cohort for the primary endpoint, and middle and rightmost boxes indicate the cohorts for the secondary endpoints.

Of 38 patients who measured their BP with both devices, 25 collected at least one overlapping week of BPs with each device, with a minimum of 12 valid daytime readings over at least 3 days per week (**Figure 1**). Of these 25 patients, 11 were female (44%), 21 were white (84%), and 23 were non-Hispanic (92%). Their average age was 59 ± 12 years. Out of 28,971 daytime measurements obtained with the cuffless wrist monitor for these 25 participants, 5,790 were overlapping with 1,300 HBPM measurements.

### Comparison of cuffless BP to HBPM

**Figure 2.**
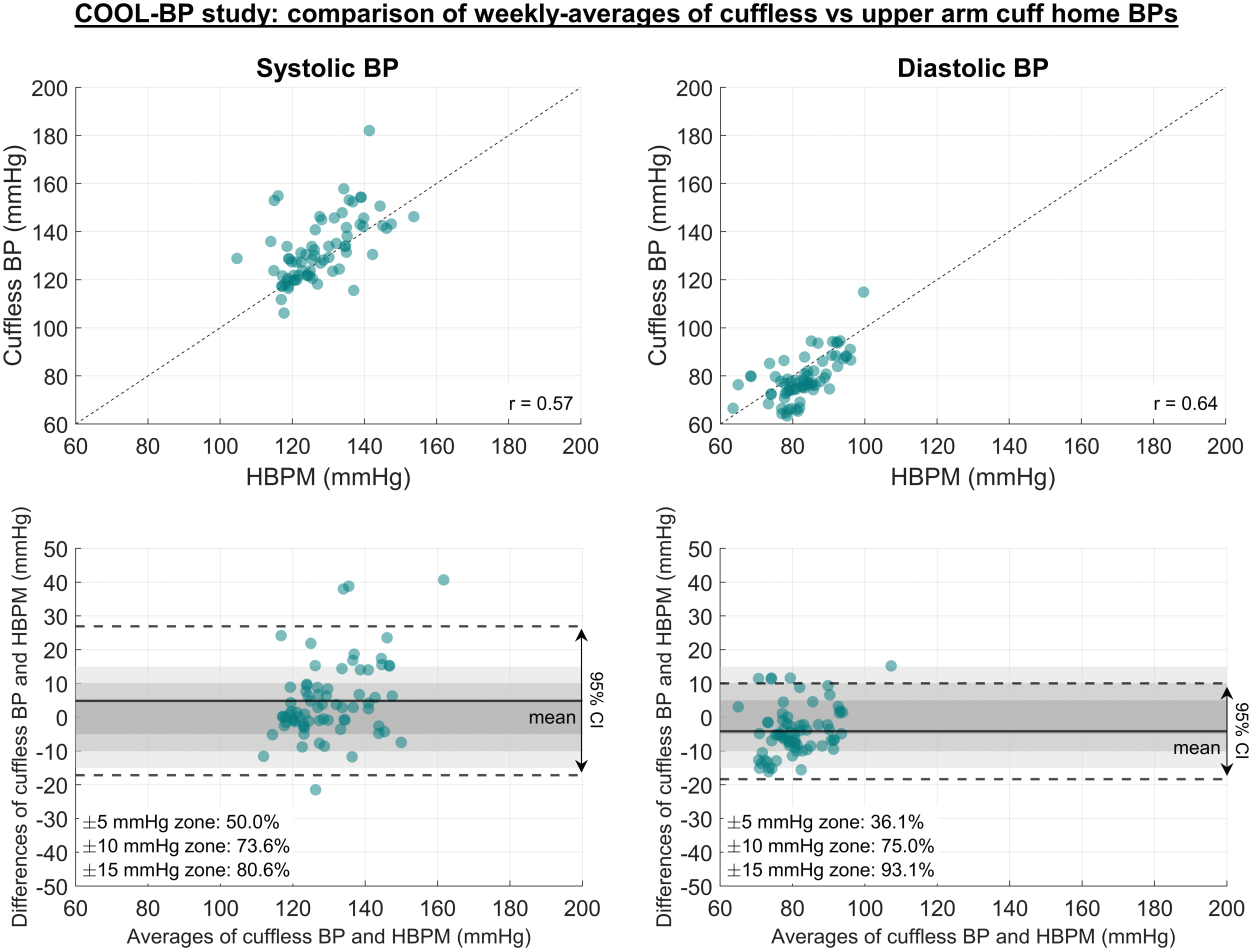
Correlation of HBPM and cuffless wrist monitor measurements. Scatter plots (upper) and Bland-Altman plots (lower) comparing weekly averages of SBP and DBP measured with HBPM and the cuffless wrist monitor. HBPM SBP and DBP (mean ± SD) were 127.6 ± 9.6 mmHg and 82.8 ± 7.3 mmHg; cuffless wrist monitor SBP and DBP (mean ± SD) were 132.5 ± 13.5 mmHg and 78.6 ± 9.2 mmHg 127.6 ± 9.6 mmHg and 82.8 ± 7.3 mmHg.

We identified 72 periods of overlapping BP measurements in the 25 patients. There was a moderate correlation between average nonsimultaneous daytime BPs by HBPM and the cuffless device (*r* = 0.57, 95% CI: 0.39–0.71 for SBP [*P* < 0.001]; *r* = 0.64, 95% CI: 0.48–0.76, for DBP [*P* < 0.001]). Cuffless device SBP (mean ± SD) was 132.5 ± 13.5 mmHg compared to traditional HBPM SBP of 127.6 ± 9.6 mmHg. Cuffless device diastolic BP (DBP) was 78.6 ± 9.2 mmHg compared to traditional HBPM DBP of 82.2 ± 7.31. Average cuffless SBP was higher than HBPM (4.9 ± 11.3 mmHg, *P* = 0.01), while DBP was lower (−4.2 ± 7.2 mmHg; *P* = 0.003) (**Figure 2**).

### Tracking changes in BP after a medication titration

**Figure 3.**
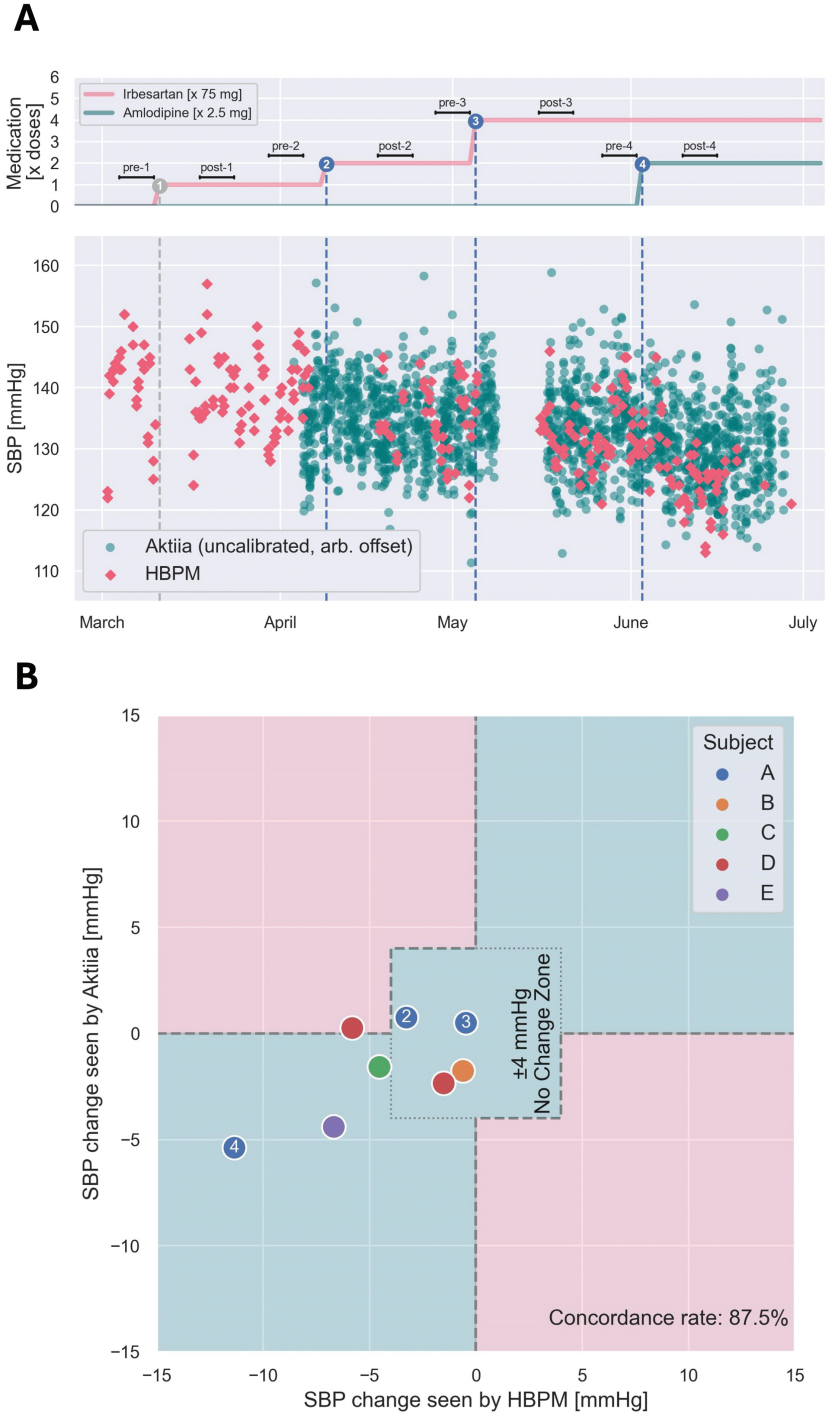
Detection of BP changes with Aktiia vs HBPM. (**a**) Visual illustration of time series of SBPs measured using HBPM and cuffless wrist monitor. A minimum 7-day drug stabilization period was required following each drug titration, during which no recalibration of the cuffless device occurred. (**b**) Concordance plot for systolic BP changes seen by HBPM and cuffless wrist monitor over the eight events for the five patients. All patients are represented by a single dot, except for Subjects A and D who are represented by three and two dots, respectively, one for each medication titration. A 4 mmHg no change zone was considered in the calculation of concordance rate, reflecting less than half the tolerable error of the cuff. Points were deemed concordant if within the middle, right-upper, or left-lower area and discordant if within left-upper or right-lower area. Numbered dots correspond to the respective medication titrations presented in panel **a** (for Subject A).

Five patients collected overlapping qualifying BP measurements with both devices over eight medication titrations. Overall, there was good concordance (87.5%) between SBP changes detected by the two devices (**Figure 3**; Supplementary Figure 1). After four of the dose changes, both HBPM and cuffless wrist monitor showed ≤4 mmHg change (no change zone). After three of the dose changes, both HBPM and cuffless wrist monitor showed a decrease in BP. HBPM and cuffless wrist monitor were discrepant after one of eight titrations, where HBPM showed a decrease and the wrist monitor showed no change/slight increase.

### Participant preferences

**Figure 4.**
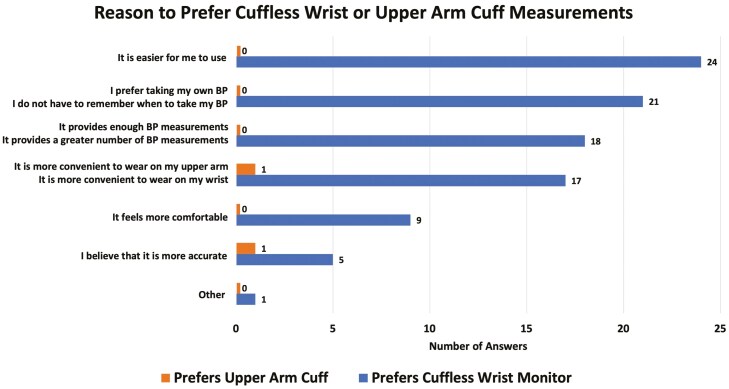
Patient preferences. Multiple answers were possible. Where participants were provided different reasons for preferring each device, they are listed in separate lines.

Of 35 patients who used both devices, 34 completed all three surveys. Thirty-one patients (91%) indicated they preferred using the cuffless wrist monitor over the upper arm BP cuff, one (3%) preferred the BP cuff, and two (6%) had no preference. Top reasons for preferring the bracelet included ease of use, convenience, and number of readings (**Figure 4**). Most patients (27/34, 79%) used the cuffless device either daily or most days of the week, and 23 (68%) found it easy or very easy to use. Twenty-eight (82%) patients believed the bracelet could help manage their HTN; 32 (94%) indicated that direct transmission of bracelet BP to their providers would be valuable (**Figure 4**).

### Adverse events

There were seven unexpected nonserious adverse events reported either directly to the study team (1) or through the patient satisfaction survey (6) completed by participants at days 30, 60, and 180 during the trial. The directly reported adverse event was a bout of contact dermatitis from wearing the bracelet, which resolved when the bracelet was removed. Participants reported wrist discomfort (3) or wrist skin irritation (4) from using the device. No participant withdrew from the study because of an adverse event.

## DISCUSSION

In this prospective open-label single-arm study to study the feasibility and efficacy of a cuffless BP monitoring device in a remote hypertension monitoring program, we report three important findings. First, there was moderate correlation between average BP measurements taken nonsimultaneously with traditional HBPM and a cuffless device. Second, there was good concordance between SBP changes detected by the two devices after a medication titration in a small number of patients. Finally, most patients regularly used the cuffless device, an overwhelming majority preferred it over cuff-based HBPM, and almost all found it easy to use. As this study was conducted as part of a remote HTN program that used HBPM for management of BP, these findings suggest that continual cuffless monitoring might be useful for the remote management of HTN, upon further validation.

Accurate and regular out-of-office BP measurements are recommended for effective HTN management.^6^ Traditionally, HBPM and ABPM have provided these data; however, both methods have significant limitations.^13^ HBPM faces several challenges, including a lack of awareness about validated devices despite availability of educational resources like validatebp.org and stridebp.org, reliance on patients to measure, record, and report BP accurately, and the potential for anxiety surrounding BP measurement. Additionally, educational support is necessary, long-term adherence is difficult, and, despite new reimbursement codes, the cost of devices is often not covered by insurance.^13^ ABPM also faces many challenges, resulting in extremely low utilization among Medicare beneficiaries. The reasons are multiple, including reimbursement policies and economic considerations.^14^ Providers encounter obstacles implementing ABPM, which requires trained staff, extra time to prepare patients, and adequate equipment and specialists for referrals.^13,15^ Finally, patient-level barriers for ABPM include low patient tolerance due to the cumbersome device and side effects ranging from bruising to sleep disturbance.^13,16^ All of these substantial challenges highlight the need for an alternative, practical approach to BP measurement.

A key outcome of our study was the correlation between BP measurements obtained from a cuffless device and HBPM. The validation of the cuffless device used in this study has been demonstrated by multiple studies under current validation standards.^7–11^ However, before routine clinical use of these devices, further validation is needed with updated ISO standards^17,18^ which are in preparation.^19^ Importantly, we did not conduct this study to add to the validation of its accuracy. Rather, we aimed to demonstrate the feasibility of using the cuffless device in a remote hypertension management program. Given the significant differences in number and timing of measurements, a certain level of discrepancy was anticipated, similar to comparisons of ABPM vs HBPM.^20^ However, our findings still revealed correlation between the two methods. The frequent and varied timing of cuffless BP measurements throughout the day provided a comprehensive profile of the patient’s BP, counterbalancing expected discrepancies due to nonsimultaneous measurements. This correlation is particularly significant as it suggests feasibility of cuffless devices to provide BP readings that are consistent with traditional methods as part of a remote program. Further exploration is warranted to add continual cuffless monitoring to the armamentarium for the management of HTN, especially in the remote setting. In addition, continual BP monitoring can be used to capture the metric of time-in-target range, which shows great promise to predict risk of cardiovascular events.^21^ It is important to note that cuffless monitors readings are based upon central rather than peripheral BP, and therefore demonstrate differences in the extent of nocturnal dipping.^9^ This difference was irrelevant in our study, as only daytime BPs were used to match the times for HBPM.

We also investigated the effectiveness of cuffless BP devices in detecting changes in BP following medication titrations. This investigation was especially important given a previous report, based on three participants, showing cuffless BP devices were unable to track BP decline accurately after a medication titration compared to HBPM.^5^ Our study, including only five patients but eight medication titration events, revealed a contrary result. It demonstrated a good concordance between cuffless device and HBPM in identifying BP changes postmedication titrations. The ability of cuffless devices to track BP changes accurately postmedication titration holds significant implications for remote HTN management. We reported titration data for cuffless BP monitors based on fewer participants than expected because of challenges with capturing overlapping BP measurements in patients wearing two separate devices before and after medication titrations. Anticipating these challenges, larger studies need to be conducted to yield more substantial set of BP measurements around medication titrations.

Cuffless BP devices are designed to be nonintrusive, easy to use, and capable of continual BP measurement. They also can capture BP measurements in different body positions, as opposed to traditional cuffed BP devices.^8^ In our study, most patients reported a remarkably high level of compliance with their devices, using them almost daily. The high usage rate is likely a reflection of a user-friendly design, and a device less cumbersome than traditional cuff-based monitors. Patients reported strong interest in being part of an HTN management program that enables automatic BP data transmission to healthcare providers to enhance a seamless process. Such programs might not only alleviate the burden of manual record-keeping from the patients but also reduce the alarm reaction leading to situationally elevated BP measurements, since patients do not need to trigger measurements and are unaware when their BP is measured.

The study presents several notable strengths and limitations. A primary strength was its prospective design, which captured real-world data from a HTN management program and patient experiences. This approach offers insights into daily BP management outside the controlled conditions of traditional clinical trials, enhancing the relevance and applicability of the findings but with more robust data collection compared to a retrospective study. Second, the study benefits from the automated transmission capabilities of both traditional and cuffless BP devices, which facilitate real-time data collection, not dependent on patient report. Finally, the incorporation of patient surveys provided valuable insights into patient perceptions and experiences, thereby enriching understanding of the practicality and acceptability of these technologies in routine use. Conversely, the nonrandomized design of the study may have introduced selection biases, impacting the validity of the results. Furthermore, the small sample size limits the generalizability of the findings to broader populations. Small sample size was compounded by 13 patients discontinuing participation. During the COOL-BP study, a significant number of patients dropped out, largely due to initial challenges with pairing or initiating the cuffless wrist device. This issue stemmed from the use of early versions of the Aktiia system by participants, which lacked enhancements found in later commercial releases that improved cuff initialization. To maintain data integrity and consistency, participants continued using the original version despite subsequent improvements made available by Aktiia. The concern for small sample size is significant given the emerging nature of cuffless BP technology, where long-term data on accuracy and reliability across diverse patient demographics are not yet available. Furthermore, we reported titration data based on fewer participants than expected because of challenges capturing overlapping BP measurements in patients wearing two separate devices before and after documented medication titrations. Anticipating these challenges, future research should aim for a randomized study design with a larger and more heterogenous patient cohort and extended duration of follow-up.

Cuffless BP devices offer a promising and innovative solution to the challenges of BP monitoring in HTN management. Their ease of use and potential for seamless data integration make them an attractive option for both patients and healthcare providers. Our study suggests that cuffless devices could significantly enhance patient compliance, data accuracy, and overall effectiveness of HTN control, particularly with remote management. Further research with larger cohorts is required for generalization of the findings and consolidation of the role these devices may play in enhancing patient-centered approaches and health outcomes in HTN management.

## Supplementary Data

Supplementary materials are available at *American Journal of Hypertension* (http://ajh.oxfordjournals.org).

hpaf003_suppl_Supplementary_Materials

## Data Availability

Individual participant data, including data dictionaries, will not be made publicly available due to the inclusion of private health information. However, data access may be granted upon request, subject to local ethics restrictions and data protection regulations.
