# Supplementary material for: Continual Versus Occasional Blood Pressure (COOL-BP) in Remote Hypertension Management
Source: Am J Hypertens. 2025 Jan 10;38(5):295–302. doi: 10.1093/ajh/hpaf003 (PMC11997243; doi:10.1093/ajh/hpaf003)
Supplement: hpaf003_suppl_Supplementary_Materials [file hpaf003_suppl_supplementary_materials.docx]

**Continual Versus Occasional Blood Pressure (COOL-BP) in Remote Hypertension Management**

**Supplemental Materials**

**Supplemental Figure 1. Time Series Illustration of Systolic Blood Pressure Measurements from HBPM and a Cuffless Wrist Monitor**

1. **Subject B**


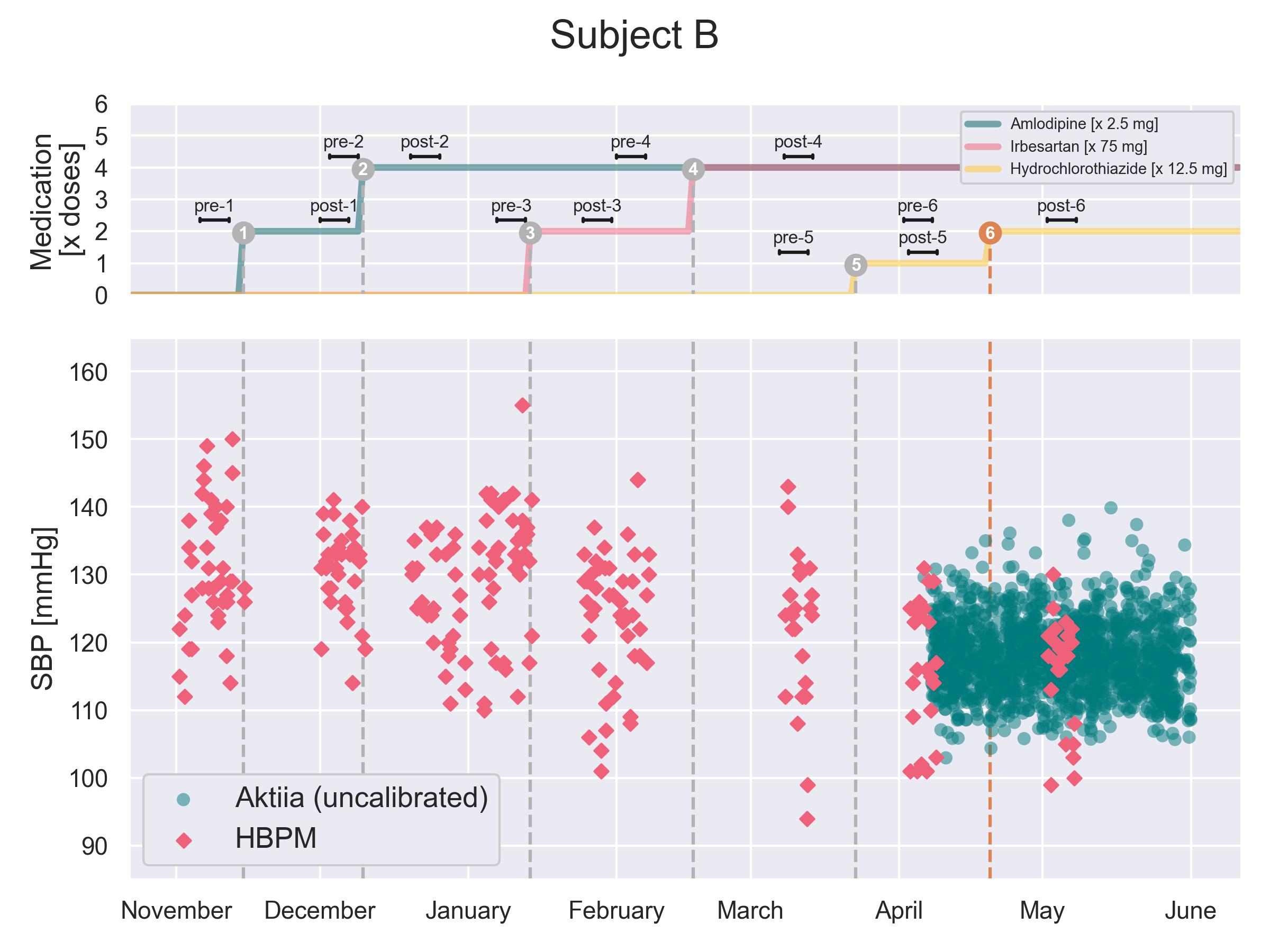


1. **Subject C**


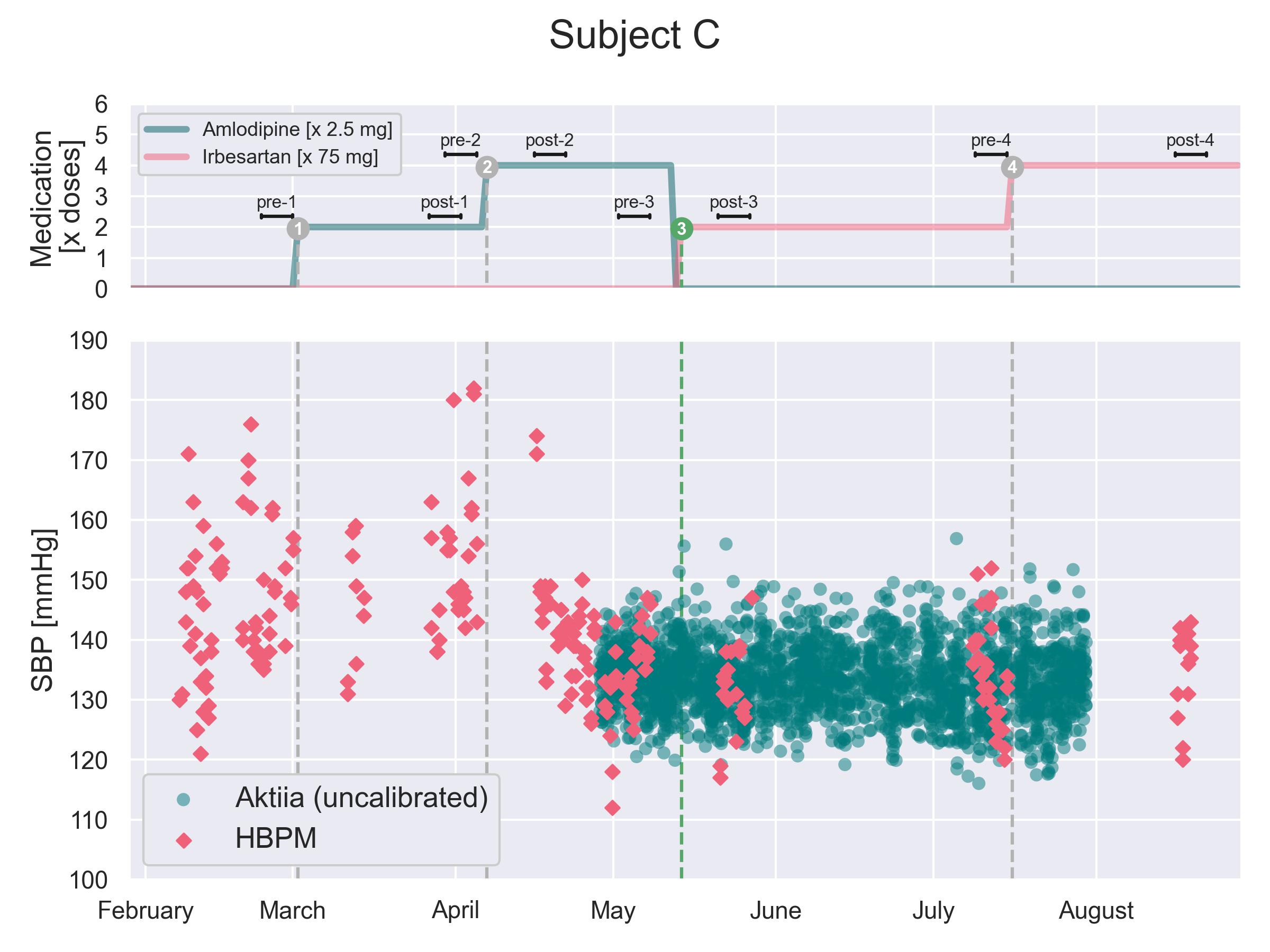


1. **Subject D**


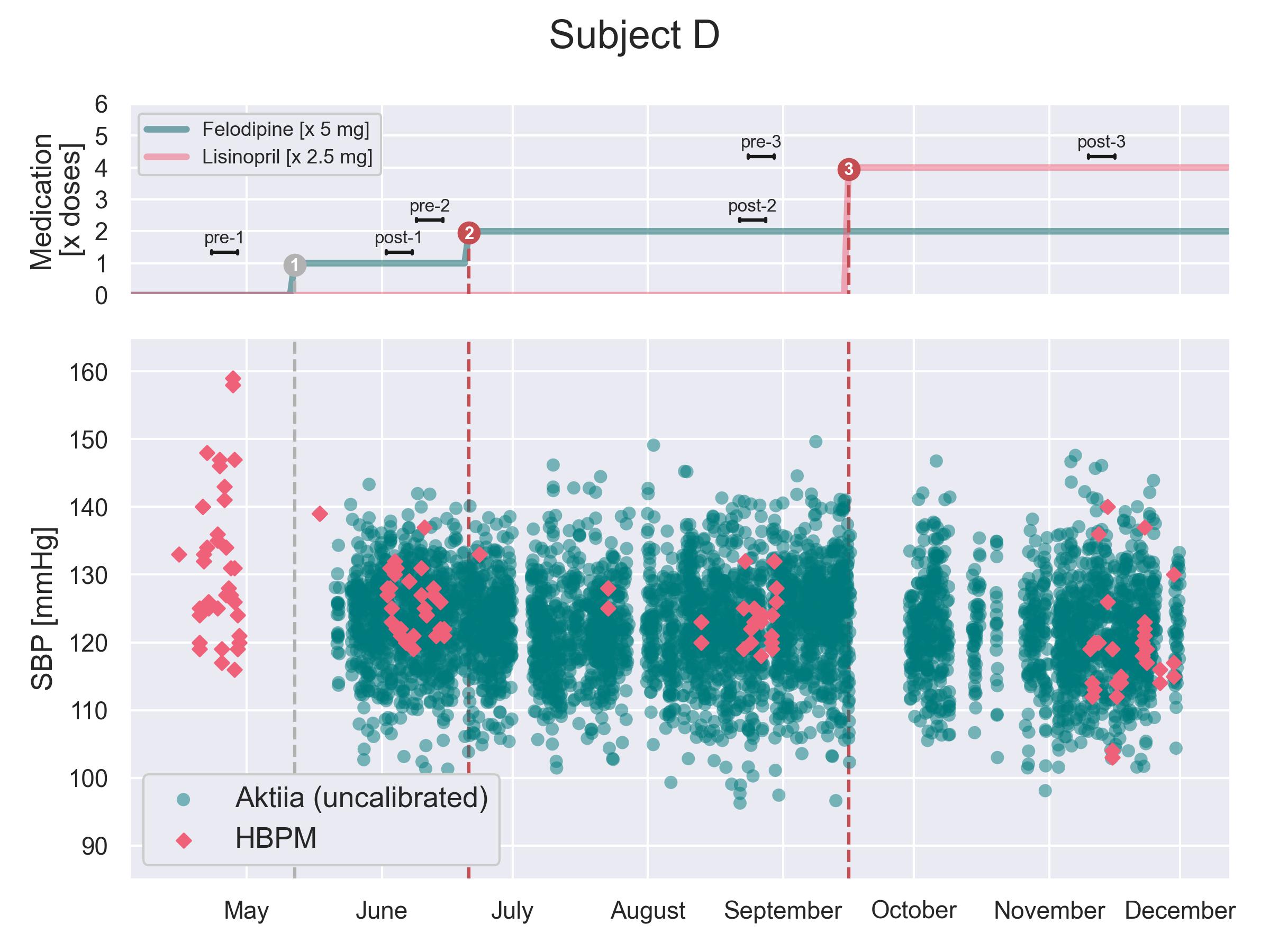


1. **Subject E**


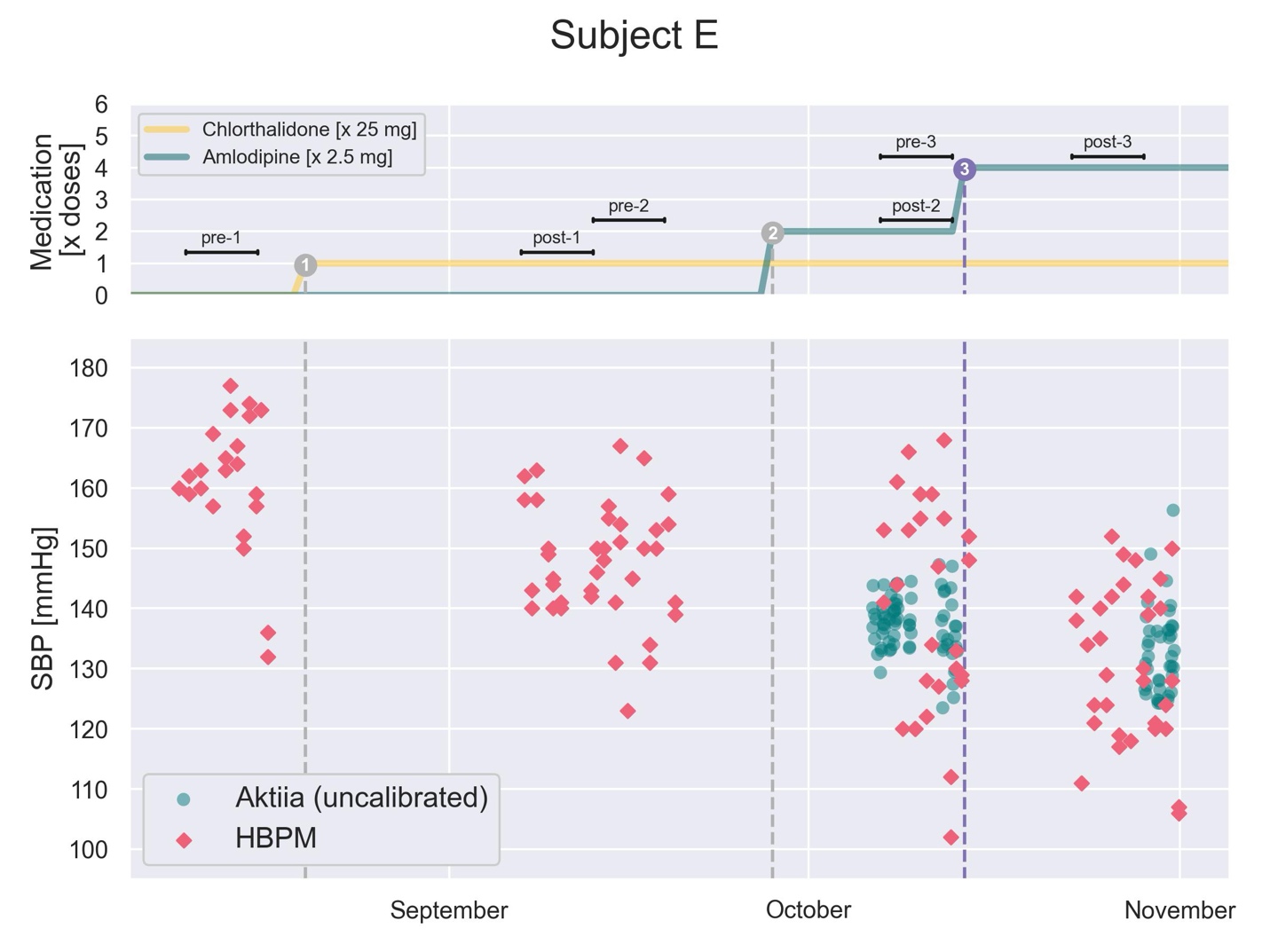


**List of medication titration dates for each patient included in medication titration analysis, Start - End (dd/mm/yyyy)**

Patient A:

- Irbesartan, 75 mg, 11/03/2022 – 08/04/2022
- Irbesartan, 150 mg, 09/04/2022 – 05/05/2022
- Irbesartan, 300 mg, 05/05/2022 – NA
- Amlodipine, 5 mg, 03/06/2022 – NA

Patient B:

- Amlodipine, 5 mg, 15/11/2021 – 10/12/2021
- Amlodipine, 10 mg, 10/12/2021 – NA
- Irbesartan, 150 mg, 14/01/2022 – 14/02/2022
- Irbesartan, 300 mg, 17/02/2022 – NA
- Hydrochlorothiazide, 12.5 mg, 23/03/2022 – 20/04/2022
- Hydrochlorothiazide, 25 mg, 20/04/2022 – NA

Patient C:

- Amlodipine, 5 mg, 02/03/2022 – 07/04/2022
- Amlodipine, 10 mg, 07/04/2022 – 12/05/2022
- Irbesartan, 150 mg, 14/05/2022 – 15/07/2022
- Irbesartan, 300 mg, 16/07/2022 – NA

Patient D:

- Felodipine, 5 mg, 12/05/2022 – 22/06/2022
- Felodipine, 10 mg, 21/06/2022 – NA
- Lisinopril, 10 mg, 16/09/2022 – NA

Patient E:

- Chlorthalidone, 25 mg, 20/08/2022 – NA
- Amlodipine, 5 mg, 28/09/2022 – 14/10/2022
- Amlodipine, 10 mg, 14/10/2022 – NA
- Irbesartan, 75 mg, 05/10/2022 – NA
